# Medial Canthal Gouty Tophus: A Report of a Rare Case

**DOI:** 10.7759/cureus.107469

**Published:** 2026-04-21

**Authors:** Zhi Yiu Hiang Weang Lim, Mohamad Aziz Salowi, Woan Shian See, Sylves Patrick, Tham Joon Hi, Hanida Hanafi

**Affiliations:** 1 Ophthalmology, Hospital Queen Elizabeth, Kota Kinabalu, MYS; 2 Ophthalmology, Ministry of Health Malaysia, Putrajaya, MYS; 3 Ophthalmology, Hospital Selayang, Selayang, MYS; 4 Ophthalmology, Faculty of Medicine and Health Sciences, Universiti Malaysia Sabah, Kota Kinabalu, MYS; 5 Pathology and Laboratory Medicine, Hospital Queen Elizabeth, Kota Kinabalu, MYS

**Keywords:** case report, eyelid mass, gout, gouty tophus, medial canthus

## Abstract

Subcutaneous collections of monosodium urate crystals, known as gouty tophi, usually develop in joints and distal parts of the limbs. Their appearance around the eye is very uncommon. We present the case of a 38-year-old Malay gentleman with a nine-year history of poorly controlled gout who developed a progressively enlarging, painless mass at the right medial canthus. Serum uric acid was 727 μmol/L (reference range: 200-420 μmol/L). Clinical examination revealed a 5 × 5 mm yellowish, nodular, nonmobile, nontender mass. Excision biopsy was performed, and histopathology showed an intradermal aggregate of acellular eosinophilic material rimmed by multinucleated giant cells, histiocytes, and lymphoplasmacytic cells, findings diagnostic of a gouty tophus. Postoperative recovery was uneventful. This case represents the first reported medial canthal gouty tophus in Malaysia. Gouty tophus should be considered in the differential diagnosis of periocular masses, especially in patients with chronic gout and hyperuricemia.

## Introduction

Gout is the most common form of inflammatory arthritis worldwide, resulting from chronic elevation of serum uric acid levels [1]. It typically presents with recurrent acute attacks of severe joint pain, swelling, and redness, most often affecting the first metatarsophalangeal joint [1]. Hyperuricemia is defined as a serum urate level exceeding 420 μmol/L (7 mg/dL) in adult males. Persistently elevated levels lead to supersaturation and precipitation of monosodium urate (MSU) crystals in tissues. When hyperuricemia persists, MSU crystals can aggregate into organized subcutaneous deposits known as “gouty tophi” [1,2]. These tophi elicit a chronic granulomatous inflammatory response, characterized histologically by a central core of MSU crystals surrounded by multinucleated giant cells, histiocytes, fibroblasts, lymphocytes, and plasma cells [3].

Tophi usually develop in sites with lower temperature, repetitive microtrauma, or reduced blood flow, such as the digits, the pinna of the ear, the olecranon bursa, and the prepatellar bursa [1,2]. Clinically, tophi present as firm, painless, yellowish nodules that slowly enlarge over months to years. They are typically nontender unless inflamed, and the overlying skin may become thin, ulcerate, or discharge a chalky material in advanced cases. However, the periocular region is an exceptionally rare location for tophus formation. The global prevalence of gout is approximately 4% in adults, and tophaceous gout develops in 10-30% of patients with poorly controlled disease. Despite this, only seven cases of periocular gouty tophi have been reported in the English literature over 35 years, giving an estimated incidence of less than 0.001% among gout patients. This extreme rarity justifies the reporting of individual cases to characterize the clinical presentation, histopathology, and optimal management of this unusual entity.

Clinically, a periocular tophus can mimic more common lesions such as an epidermoid cyst, chalazion, sebaceous cyst, or even a neoplasm. The differential diagnosis includes epidermoid cyst, chalazion, dermoid cyst, xanthelasma, and basal cell carcinoma. Therefore, a high index of suspicion is required, especially in patients with a known history of poorly controlled gout or persistent hyperuricemia.

In this report, we present a rare case of a medial canthal gouty tophus in a young man with long-standing, untreated gout. We discuss the clinical presentation, histopathological features, and the importance of considering systemic metabolic diseases in the differential diagnosis of periocular masses.

## Case presentation

A 38-year-old Malay gentleman with a nine-year history of gout presented to our ophthalmology clinic with a painless swelling over the right medial canthus. He first noticed the lesion approximately one year prior to presentation. Initially small and barely noticeable, the swelling had been slowly but progressively increasing in size over the subsequent months. Initially estimated at 2 mm, the mass grew to 5 mm × 5 mm over 12 months. He denied any associated pain, tenderness, itching, bleeding, or ulceration from the lesion. There was no history of trauma, insect bite, or previous surgery in the periocular area. He also reported no similar swellings elsewhere on his body.

The patient had a long-standing history of gout, diagnosed nine years earlier based on typical acute arthritis episodes and elevated serum uric acid. However, he had defaulted from rheumatology follow-up for the past four years and had not been taking any urate-lowering therapy (e.g., allopurinol or febuxostat) or anti-inflammatory medications for prophylaxis. He cited lack of symptoms between attacks and financial constraints as reasons for nonadherence.

Over the preceding year, he experienced more than 20 acute gout attacks, predominantly affecting his great toes, ankles, and knees. The attacks were characterized by severe pain (rated 8-9/10), marked swelling, and erythema. Each attack typically lasted three to five days and partially responded to oral diclofenac obtained from a private clinic without regular prescription renewal, which he took intermittently (reducing pain to 4-5/10). Systemic review was otherwise unremarkable. He denied fever, weight loss, night sweats, or other constitutional symptoms. There was no history of nephrolithiasis, hematuria, or intercritical arthralgia.

Baseline investigations showed serum creatinine of 82 μmol/L, eGFR >90 mL/min/1.73 m², and urine dipstick negative for protein, blood, and glucose, indicating preserved renal function with no evidence of urate nephropathy or nephrolithiasis. The patient was a frequent defaulter from medical follow-up for the past nine years and did not have proper laboratory records prior to this presentation. Therefore, no historical laboratory data were available for comparison. Full blood count, liver function tests, and lipid profile were within normal limits. Inflammatory markers such as ESR and CRP were not measured, as the patient was not experiencing an acute gout flare at the time of presentation.

Ophthalmic examination revealed a best-corrected visual acuity of 6/6 in both eyes. Pupils were equal, round, and reactive to light, with no relative afferent pupillary defect. Extraocular movements were full in all directions. Slit-lamp examination of the anterior and posterior segments was unremarkable.

**Figure 1:**
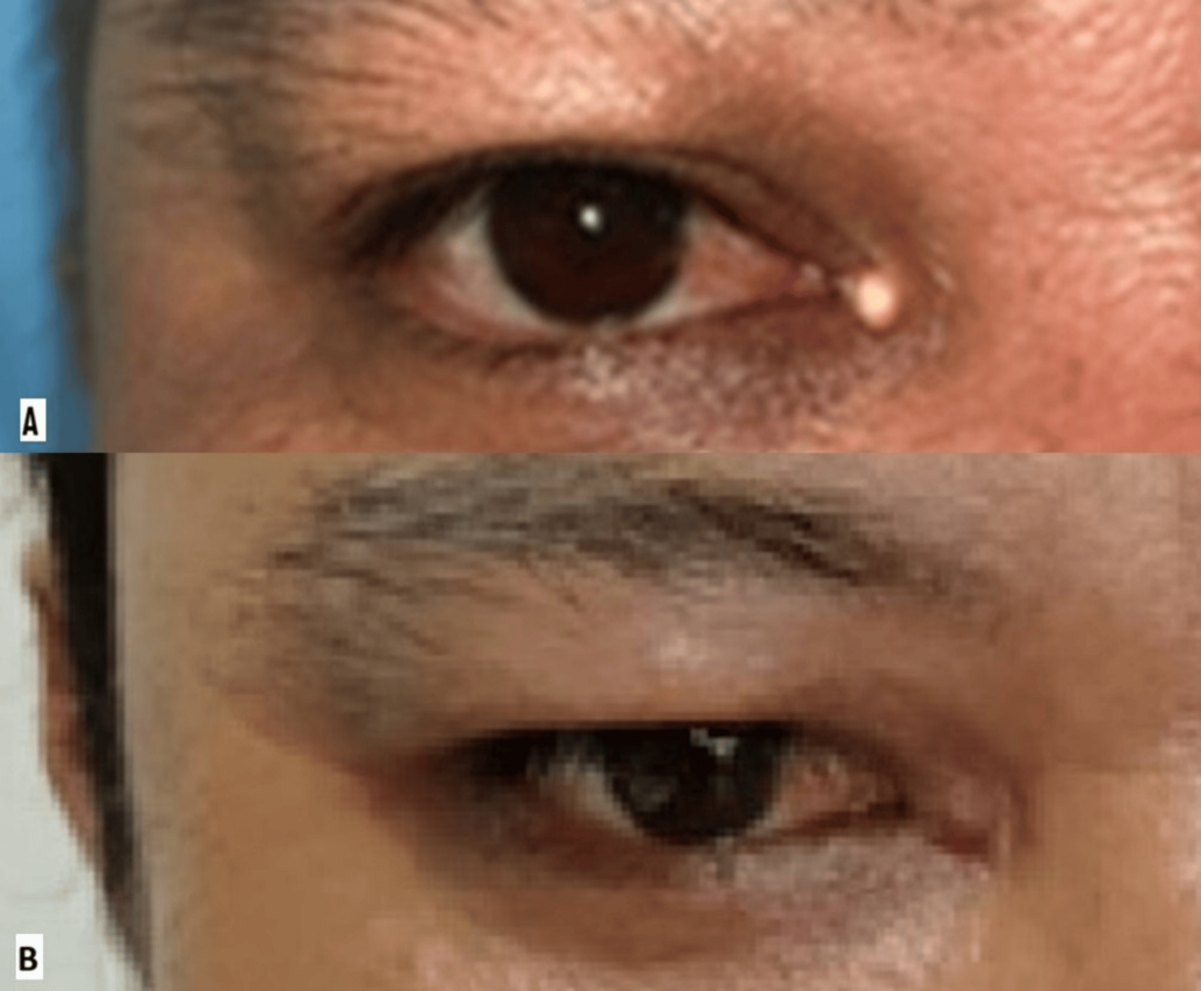
Clinical appearance of the right medial canthal mass (A) A yellowish, nodular mass measuring 5 × 5 mm at presentation. (B) Postoperative appearance at one month after excision biopsy showing a well-healed surgical site with minimal scarring.

At the right medial canthus, there was a solitary, yellowish, nodular mass measuring approximately 5 mm × 5 mm (Figure 1A). The lesion was firm, nonmobile, and nontender to palpation. Its surface was smooth, and the overlying skin was intact with no erythema, ulceration, or telangiectasia. The mass did not transilluminate, and there was no regional lymphadenopathy.

Laboratory investigations performed on the day of presentation showed a serum uric acid level of 727 μmol/L (reference range: 200-420 μmol/L). Hyperuricemia is defined as a serum urate level exceeding 420 μmol/L in adult males. Levels above 480 μmol/L are classified as severe. The patient’s level of 727 μmol/L, therefore, represents severe hyperuricemia, which correlates with his clinical history of >20 acute attacks per year and the presence of palpable tophi at multiple sites.

Based on the clinical suspicion of a gouty tophus, an excisional biopsy of the right medial canthal mass was performed under local anesthesia. The lesion was excised with a 1 mm clear margin, and the wound was closed primarily with 7-0 nylon sutures. The procedure was uneventful, and the patient tolerated it well. He was referred to rheumatology for long-term management, including re-initiation of urate-lowering therapy with dose titration to achieve a target serum urate <360 μmol/L, prophylaxis against acute flares during treatment initiation, dietary counseling, and monitoring of renal function and cardiovascular risk factors.

**Figure 2:**
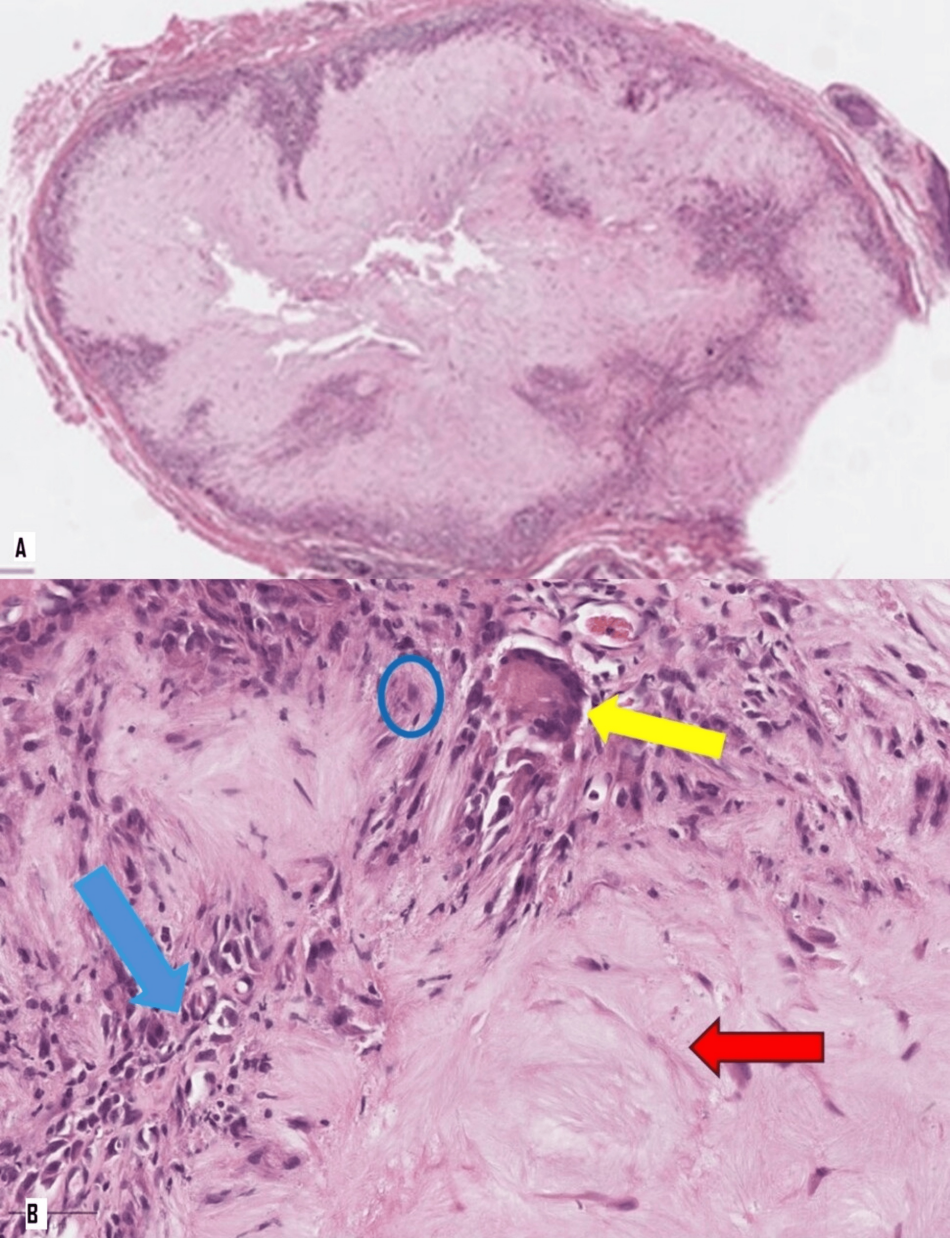
Histopathological findings of the excised mass (A) Overview of the nodular, intradermal lesion (H&E, ×20). (B) Higher magnification showing acellular, amorphous, pale eosinophilic material (red arrow) rimmed by lymphoplasmacytic infiltrate and histiocytes (blue circle), with a multinucleated giant cell (yellow arrow) (H&E, ×400).

Histopathological examination revealed eyelid skin with an intradermal mass composed of acellular, amorphous, pale eosinophilic material rimmed by multinucleated giant cells, histiocytes, and lymphoplasmacytic cells (Figure 2A, 2B), consistent with a gouty tophus.

At the one-month follow-up, the surgical site had healed well with minimal scarring (Figure 1B), with no local recurrence. At 12 months, there was no clinical evidence of tophus recurrence at the medial canthus. Serum uric acid levels showed a declining trend under rheumatology care: from 727 μmol/L at baseline to 415 μmol/L (still above target but significantly improved). The frequency of acute gout attacks reduced from >20 per year to only three attacks in the following year. He remains under ongoing follow-up.

## Discussion

Gout is a chronic inflammatory arthritis characterized by the deposition of MSU crystals in tissues [1]. The primary risk factor for gout is elevated serum urate levels [1]. Clinical manifestations of gout include acute arthritis, chronic gouty arthritis, tophaceous gout, renal impairment, and urolithiasis [2].

Gouty tophi are subcutaneous deposits of urate that form granulomas containing MSU crystals [2]. Histologically, these tophi typically exhibit a crystalline core surrounded by multinucleated giant cells (foreign body type), histiocytes, fibroblasts, lymphocytes, and plasma cells [3]. MSU crystals are water soluble and dissolve during routine processing, which is why they are often not seen on standard H&E staining; however, special anhydrous processing can be used, which will show MSU crystals that are birefringent under polarized light, confirming the diagnosis. Locations for tophi include intra-articular, periarticular, and extra-articular sites such as the digits of the hands and feet, knees, and the olecranon bursa [2]. Rare sites include the periocular area and the heart [2]. To the best of our knowledge, only seven cases of gouty tophi in the periocular area have been reported, with two cases in the medial canthal region, three in the lateral canthal region, and two in the upper eyelid region, as summarized in Table 1 [4-10]. The tendency for urate accumulation at tendons may explain the higher frequency of tophi formation in the canthal regions [10].

**Table 1:** Case reports of eyelid tophi

Author	Age (years)	Sex	Duration of gout (years)	Duration of lesion	Location of the lid lesion	Lesion size (mm)	Other lesions
De Monteynard et al. [4]	62	F	-	Two days	Lateral canthus	-	-
Morris and Fleming [5]	44	M	-	One year	Lateral canthus	6 × 5 × 4	-
Jordan et al. [6]	68	M	20	Two years	Medial canthus	5 × 6 × 4	Elbow
Yang et al. [7]	64	M	-	Nine years	Middle upper eyelid	14 × 10 × 8	Fingers
Nakatsuka et al. [8]	41	M	10	One year	Lateral canthus	4 × 7 × 4	Ankle, first metatarsophalangeal
Ing and Philteos [9]	41	M	Three	One year	Temporal upper lid	4 × 5 × 4	Knee? (no surgical specimens were sent for pathology for gout)
Chu et al. [10]	27	M	Three	Three months	Medial canthus	11 × 5 × 5	First metatarsal

In our case, the inflammatory changes observed were nodular aggregates of acellular and pale eosinophilic material rimmed by multinucleated giant cells. Light microscopy revealed needle-shaped MSU crystals, which are strongly negatively birefringent. For optimal visualization, tissue samples should ideally be fixed and stained using nonaqueous methods because urate is water-soluble. Unfortunately, routine tissue processing for histopathological examination uses aqueous solutions, which dissolve the uric acid crystals. Consequently, the needle-shaped MSU crystals were only vaguely visible within the eosinophilic material in our case.

## Conclusions

We report the first documented case of a medial canthal gouty tophus in Malaysia. This case highlights that gouty tophi, although typically found in peripheral joints and soft tissues, can rarely involve the periocular region, including the medial canthus. Clinicians should maintain a high index of suspicion for tophaceous deposits when evaluating a painless, yellowish, nodular periocular mass, especially in patients with a long-standing history of poorly controlled gout or persistent hyperuricemia. The diagnosis of a periocular gouty tophus cannot be made on clinical grounds alone. Excisional biopsy with histopathological examination remains the gold standard for definitive diagnosis. Characteristic microscopic findings, acellular, amorphous eosinophilic material surrounded by a granulomatous reaction containing multinucleated giant cells, confirm the diagnosis, even when urate crystals are dissolved by routine aqueous processing.

This case also underscores the importance of systemic evaluation and referral for long-term urate-lowering therapy. Untreated tophaceous gout can lead to progressive joint destruction, renal impairment, and cardiovascular morbidity. Therefore, ophthalmologists who identify a periocular tophus should not only manage the local lesion but also advocate for comprehensive metabolic control in collaboration with rheumatology. In summary, the medial canthus is a rare but possible location for gouty tophus. Recognition of this entity prevents misdiagnosis and unnecessary interventions while promoting appropriate systemic management of hyperuricemia.
